# Anti-Obesity Drug Orlistat Alleviates Western-Diet-Driven Colitis-Associated Colon Cancer via Inhibition of STAT3 and NF-κB-Mediated Signaling

**DOI:** 10.3390/cells10082060

**Published:** 2021-08-11

**Authors:** Bo-Ram Jin, Hyo-Jung Kim, Seo-Ah Sim, Minho Lee, Hyo-Jin An

**Affiliations:** 1 Department of Pharmacology, College of Korean Medicine, Sangji University, 83 Sangjidae-gil, Wonju-si 26339, Gangwon-do, Korea; wlsqh92@gmail.com (B.-R.J.); hyojung_95@naver.com (H.-J.K.); dusdl404@naver.com (S.-A.S.); 2 Department of Life Science, Dongguk University-Seoul, 32 Dongguk-ro, Ilsandong-gu, Goyang-si 10326, Gyeonggi-do, Korea

**Keywords:** colitis-associated colon cancer (CAC), NF-κB, STAT3, azoxymethane (AOM)/dextran sulfate sodium (DSS) model, Western diet, orlistat

## Abstract

Many researchers have argued that Western diet (WD)-induced obesity accelerates inflammation and that inflammation is a link between obesity and colorectal cancer (CRC). This study investigated the effect of WDs on the development and progression of colitis-associated colon cancer (CAC) and the efficacy of the anti-obesity agent orlistat on WD-driven CAC in mice. The results revealed that the WD exacerbated CAC in azoxymethane (AOM)/dextran sulfate sodium (DSS)-induced mice, which showed increased mortality, tumor formation, and aggravation of tumor progression. Furthermore, WD feeding also upregulated inflammation, hyperplasia, and tumorigenicity levels through the activation of STAT3 and NF-κB signaling in an AOM/DSS-induced mouse model. In contrast, treatment with orlistat increased the survival rate and alleviated the symptoms of CAC, including a recovery in colon length and tumor production decreases in WD-driven AOM/DSS-induced mice. Additionally, orlistat inhibited the extent of inflammation, hyperplasia, and tumor progression via the inhibition of STAT3 and NF-κB activation. Treatment with orlistat also suppressed the β-catenin, slug, XIAP, Cdk4, cyclin D, and Bcl-2 protein levels in WD-driven AOM/DSS-induced mice. The results of this study indicate that orlistat alleviates colon cancer promotion in WD-driven CAC mice by suppressing inflammation, especially by inhibiting STAT3 and NF-κB activation.

## 1. Introduction

Colorectal cancer (CRC) is one of the most common gastrointestinal malignant cancers and is a leading cause of cancer-related mortality across the globe [1]. This malignancy is a serious complication of inflammatory bowel disease (IBD), and the risk of CRC is related to the severity, extent, and duration of IBD [2]. Population-based studies have demonstrated that the incidence of cancer is increased by chronic inflammatory disorders [3]. It has been also reported that 5-acetylsalicylic acid (5-ASA), a medication used to treat IBD, represses the progression of ulcerative colitis (UC) to CRC, suggesting a promising therapeutic approach to anti-inflammatory drugs for CRC treatment [4]. Overshadowed by its efficacy, the adverse reactions of 5-ASA comprise diarrhea, headache, nausea and vomiting [5]. Meanwhile, chemotherapy drugs, including camptothecin (CPT), are most often used to treat CRC. CPT selectively inhibits the DNA topoisomerase type 1 and exerts antitumor activity [6]. However, the use of this compound also causes diarrhea, neutropenia and cytotoxicity [7]. Therefore, it is essential to develop new agents with low risk and toxicity.

Chronic inflammation in obese subjects has been reported by several studies, and it has been shown that the prevalence of CRC increases with obesity [8]. Epidemiological studies have suggested that severe obesity is associated with an increased risk of death in CRC patients [9]. Many studies have shown that Western diet (WD)-induced obesity accelerates inflammation and have implicated inflammation as a link between WD-induced obesity and CRC [10]. Some evidence also suggests that WD consumption destroys the intestinal mucosal barrier and induces the development of UC and CRC [11,12]. In this study, we used available experimental models to investigate whether the WD-mediated inflammatory microenvironment led to the development and progression of CRC via a novel pathway involving the activation of signal transducer and activator of transcription 3 (STAT3) and nuclear factor-kappa B (NF-κB).

NF-κB and STAT3 interact in several different ways to boost tumor-associated inflammation and consequently suppress antitumor immune responses. These factors promote tumor growth, progression, and metastasis [13]. In particular, NF-κB, a key regulator of the immune response, has been linked to tumor initiation and progression through improper immune development and inflammation via the regulation of related target genes [14]. STAT3, regarded as an important regulator of cell cycle progression, proliferation, migration, and cell survival, was markedly elevated in patients with colitis-associated colon cancer (CAC), and STAT3 phosphorylation actively induces the antiapoptotic proteins Bcl-2 and Bcl-xL, which are also correlated with tumor invasion and metastasis in CAC [15].

Orlistat is a drug registered for the treatment of obesity in several countries. It suppresses the action of gastrointestinal lipases, thereby impairing the metabolism of lipids in the gastrointestinal lumen, which can prevent the absorption of 30% of the lipids in dietary fat [16,17]. Orlistat is very hydrophobic and binds covalently to the active serine residues of pancreatic lipase. Orlistat appears to remain in the intestinal lumen with little absorption, but the drug passes through cell membranes sufficiently and exerts intracellular effects [18]. Interestingly, orlistat is known to inhibit the synthesis of fatty acid synthase (FAS) enzymes, which increase tumor growth [19]. For this reason, the antitumor activities of orlistat have been investigated in many cancer cell types, including colorectal, prostate, and hepatoma cells [20,21,22]. However, one animal study has suggested that orlistat may be associated with an increased risk of CRC. However, no such association has been reported in humans [23]. Despite this controversy, the properties of orlistat have led researchers to speculate that it might behave as an antitumor agent in the colonic tissues of mice with CAC. To date, the detailed in vivo biological efficacies of orlistat and the underlying molecular mechanisms associated with its effects on CAC have not yet been studied. Therefore, we investigated the antitumor properties of orlistat and its associated molecular mechanisms, which may involve the regulation of transcription factors in the colonic mucosa, under a WD-driven inflammatory environment and CRC conditions.

## 2. Materials and Methods

### 2.1. Experimental Animals and Sample Treatment

Male BALB/c mice (*n* = 77; 8 weeks old; 18–20 g, Daehan Biolink Co., Daejeon, Korea) were housed under conditions that were in accordance with the guidelines for the care and use of laboratory animals adopted and promulgated by the Institutional Animal Care and Use Committee (IACUC) of Sangji University (IACUC Animal approval protocol #2016-12). The mice were housed in the animal room with 12 h dark/light cycles and constant conditions (22.5 ± 2.5 °C temperature; 50 ± 10% humidity). Mice were fed a normal diet (NIH-41 open formula diet, 5% fat by calories) or a WD (D12451 open formula diet, 45% fat by calories). Using a blinded method, the animals were randomly allocated into the following groups (*n* = 11): Group 1, Con group (normal mice fed normal diet); Group 2, WD group (normal mice fed WD); Group 3, AOM/DSS group (AOM/DSS-induced CAC mice fed normal diet); Group 4, WD + AOM/DSS group (AOM/DSS-induced CAC mice fed WD); Group 5, 5-ASA group (AOM/DSS-induced CAC mice fed WD, treated with 5-ASA 75 mg/kg/day; p.o.); Group 6, CPT group (AOM/DSS-induced CAC mice fed WD, treated with CPT 1.0 mg/kg/day; i.p.); and Group 7, orlistat group (AOM/DSS-induced CAC mice fed WD, treated with orlistat 10 mg/kg/day, p.o.).

### 2.2. Induction of the CAC Model

The CAC model was established by intraperitoneally injecting mice with 12.5 mg/kg body weight of AOM dissolved in PBS. After 7 days, the mice were fed 1% DSS in drinking water for 7 days. On day 14, DSS drinking was discontinued, and 5-ASA, CPT and orlistat were given to mice every day for a week, using a blinded method. Mice were subjected to two further cycles with 2% DSS (Supplementary Scheme S1). Mice were euthanized by cervical dislocation 56 days after the first treatment with AOM, and blood and colon tissues were collected from them.

### 2.3. Histopathology

Colon tissues were fixed in 10% neutral buffered formalin and embedded in paraffin. Then, the tissue samples were cut into 5 μm sections and stained with hematoxylin and eosin (H&E). The parameters used to score the degrees of hyperplasia, inflammation and tumorigenicity are presented in Table 1, Table 2 and Table 3. The extent of histological pathology was evaluated by a medical technologist.

### 2.4. Immunohistochemical Staining

All immunohistochemical staining (IHC) was performed using 10% neutral buffered formalin-fixed, paraffin-embedded samples. The immunostaining was conducted using anti-NF-κB p65 (#4764, Cell Signaling, Danvers, MA, USA) and anti-p-STAT3 Tyr705 (#9145, Cell signaling) primary antibodies. The IHC staining was performed as previously described [24], and the slides were visualized using an optical microscope (Leica, Wetzlar, Germany). The scores of immune reactivity were determined as follows: mild, 1; moderate, 2; intense, 3; severe, 4.

### 2.5. Western Blot Analysis

Protein extracts were isolated from the colon tissues (30 mg) using the Pro-prep™ (Intron biotechnology Inc., Gyeonggi-do, Korea). The extracted protein samples (30 μg) were separated on an 8–12% sodium dodecyl sulfate–polyacrylamide gel and transferred to an immobilon-P PVDF membrane (IPVH00010, Millipore, MA, USA), as described previously [25]. The membrane was incubated with 2.5% skimmed milk for 30 min at 22–25 °C and then probed overnight with a primary antibody (dilution 1:1000 in Tween 20/Tris-buffered saline) at 4 ℃. Primary antibodies against Bcl-2 (sc-7382), Cdk4 (sc-23896), IκB-α (sc-203), STAT3 (sc-482), XIAP (sc-55552), and β-actin (sc-81178) were purchased from Santa Cruz Biotechnology, Inc. (Dallas, TX, USA). The p65 (#4764), pIκB-α (#9246), p-STAT3 Tyr705 (#9145), and p-STAT3 Ser727 (#9134) antibodies were purchased from Cell Signaling Technology (Danvers, MA, USA). After washing three times with Tween 20/Tris-buffered saline, the membrane was incubated with horseradish peroxidase-conjugated secondary antibody (Jackson ImmunoResearch, West Grove, PA, USA, dilution, 1:2000) for 2 h at 22–25 °C. The blots were visualized using enhanced chemiluminescence (Ab signal, Seoul, Korea).

### 2.6. Statistical Analyses

All the data are expressed as the mean ± S.D. of triplicate experiments (technical triplicates). The data were analyzed by one-way analysis of variance (ANOVA) followed by Dunnett’s post hoc test, and *p*-values < 0.05 were considered to be statistically significant.

## 3. Results

### 3.1. WD Consumption Exacerbated the Pathogenesis of CAC in Mice

**Figure 1:**
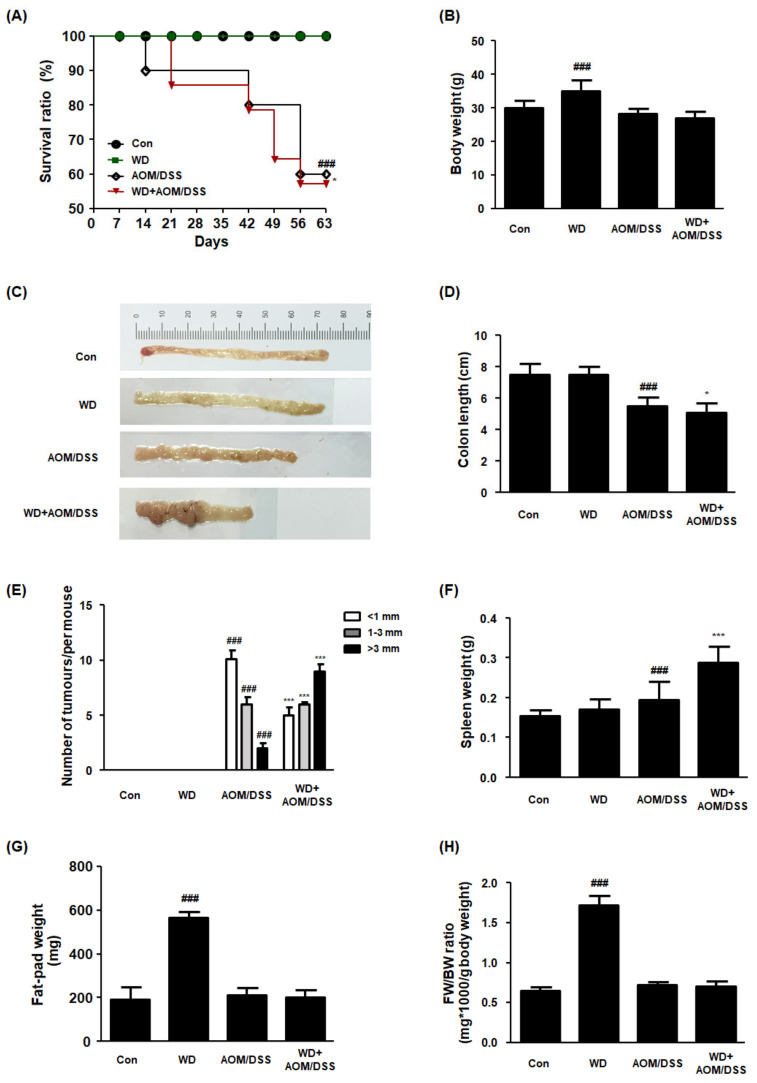
Effect of WD consumption on the development of CAC in an AOM/DSS-induced CAC mouse model. All mice were allocated to four groups: control mice (Con), Western-diet-fed mice (WD), AOM/DSS-induced CAC mice (AOM/DSS), and AOM/DSS-induced CAC mice fed WD (WD + AOM/DSS). (**A**) Survival ratio. Data were analyzed by Kaplan–Meier survival analysis (*n* = 11); (**B**) body weights for each mouse group; (**C**) representative photographs of colon tissues from each group; (**D**) colon lengths; (**E**) number of macroscopic tumors. The colon length was estimated from the cecum to the proximal rectum. All multiple nodular masses were counted as a tumor in colonic tissues in a blinded manner. (**F**) Splenic weight; (**G**) fat-pad weight; (**H**) FW/BW ratio. FW/BW ratio = fat pad weight in each mouse (mg) × 1000/body weight for each mouse (**G**). The values are the means ± SD (*n* = 6); ^###^ *p* < 0.001 when compared to Con; * *p* < 0.05 and *** *p* < 0.001 when compared to AOM/DSS.

In this study, we established a WD-fed CAC mouse model to determine the role of WD consumption in the development and progression of AOM/DSS mouse models with CAC. It can be seen from the data in Figure 1A–D that the WD + AOM/DSS group had significantly reduced survival rates, body weights, and colon lengths compared to the AOM/DSS group. In addition, the WD + AOM/DSS group showed multiple nodular masses in their colon tissues compared to the AOM/DSS group, implying that WD consumption elevated susceptibility to tumorigenesis (Figure 1E). Interestingly, the spleen weight was higher in the WD + AOM/DSS group than in the AOM/DSS group (Figure 1F). These results are consistent with those of other studies and suggest that WD feeding exacerbates inflammation and increases the risk of CRC. There were no significant differences between the AOM/DSS and WD + AOM/DSS groups in terms of fat pad weight (Figure 1G,H). In addition, there was no significant difference in the food intake between each experimental group (data not shown).

### 3.2. WD Feeding Drove CAC in Mice via Upregulation of STAT3 and NF-κB 

**Figure 2:**
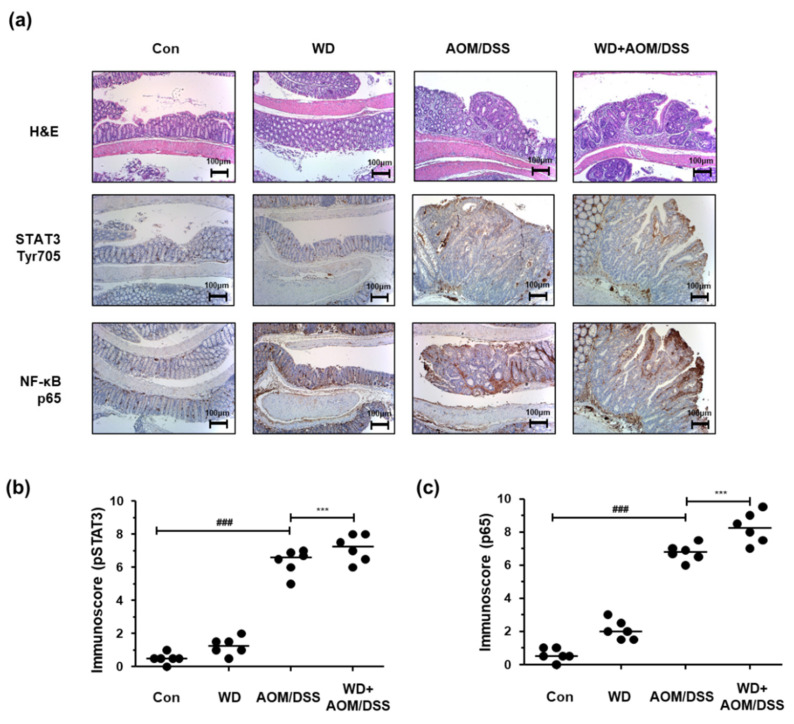
Effect of WD consumption on protein expression of NF-κB and STAT3 in an AOM/DSS-induced CAC mouse model. (**a**) Colon tissue sections from each group stained by H&E and for NF-κB p65 and STAT3 Tyr705 (original magnification, 100×); (**b**) immunoscores for STAT3 Tyr705; (**c**) immunoscores for NF-κB p65. The values are the means ± SD (*n* = 6); ^###^ *p* < 0.001 when compared to Con; *** *p* < 0.001 when compared to AOM/DSS.

We performed H&E and IHC staining to investigate the molecular mechanisms associated with the effects of WD feeding on AOM/DSS-induced CAC mice. In the AOM/DSS group, inflammation, hyperplasia, and tumor progression notably increased compared to those in the Con group. In addition, the inflammation, hyperplasia, and tumorigenicity levels were higher in the WD + AOM/DSS group than in the AOM/DSS group. We also found that the STAT3 and NF-κB transcription factors were significantly activated by AOM/DSS exposure, and that STAT3 and NF-κB activation was further intensified by WD feeding (Figure 2 and Table 4, Table 5 and Table 6).

### 3.3. Treatment with Orlistat Alleviated Development of CRC in WD-Driven CAC Mice

**Figure 3:**
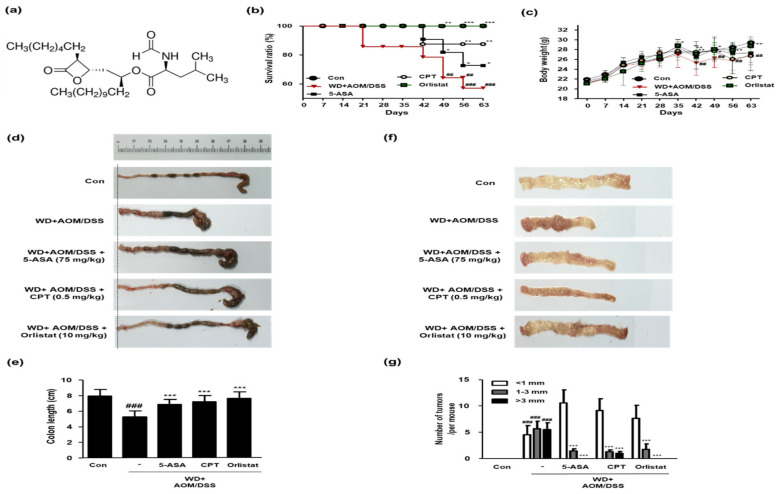
Effect of orlistat on the development of CAC in WD-driven CAC mice. (**a**) Orlistat structure; (**b**) survival ratio for each group. Data were analyzed by Kaplan–Meier survival analysis (*n* = 11); (**c**) body weight for each group (*n* = 11); (**d**) representative photographs of colonic lengths from each experimental group. (**e**) Colon length in each animal was measured between the cecum and proximal rectum. (**f**) Representative photographs of numbers of tumors in each experimental group; (**g**) number of tumors in each group. Results are the means ± SD (*n* = 6); ^##^ *p* < 0.01 and ^###^ *p* < 0.001 when compared to Con; * *p* < 0.05, ** *p* < 0.01 and *** *p* < 0.001 when compared to the WD + AOM/DSS.

In this study, we estimated the therapeutic effects of orlistat (structure shown in Figure 3a) on WD-driven CAC mice. As shown in Figure 3b, the WD + AOM/DSS group had a low survival ratio, whereas treatment with ASA, CPT, and orlistat significantly increased the survival rate of the WD-driven CAC mice. In the WD + AOM/DSS group, body weights were also significantly reduced compared to those in the Con group. However, only the orlistat treatment normalized the body weight in WD-driven CAC mice (Figure 3c). Likewise, the colon length was shorter in the WD + AOM/DSS group than in the Con group. However, treatment with ASA, CPT, and orlistat significantly increased the colon length (Figure 3d,e). Figure 3f,g shows that tumor multiplicity was significantly increased by WD feeding in the AOM/DSS mouse model, whereas treatment with ASA, CPT, and orlistat significantly reduced the number of tumors in WD-driven CAC mice.

### 3.4. Treatment with Orlistat Repressed the Tumorigenesis of Colon Tissues in WD-Driven CAC Mice

**Figure 4:**
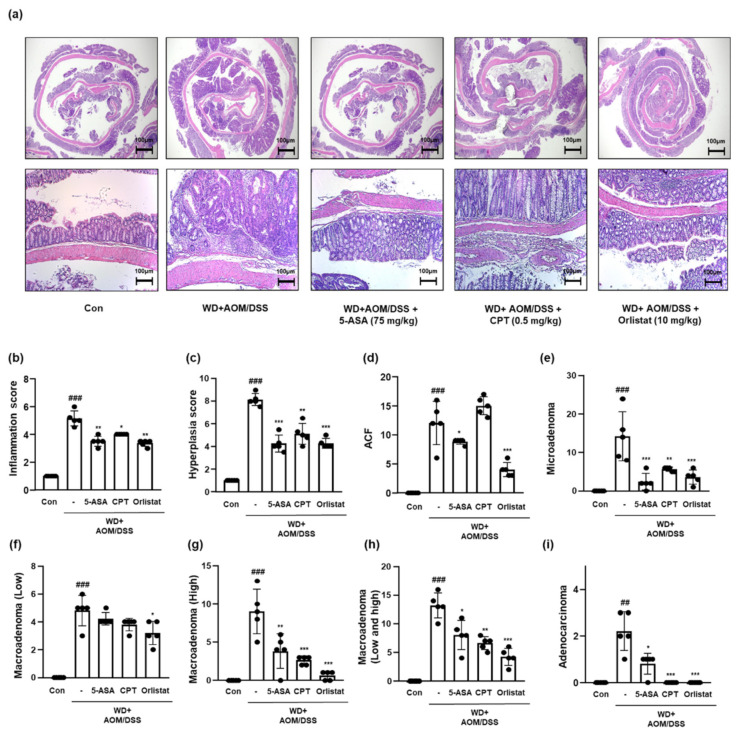
Effect of orlistat on the histological pathology of CAC in WD-driven CAC mice. (**a**) H&E staining was conducted using colon tissues from each group. Slide sections were scrutinized by microscopy. (**b**) Inflammation scores; (**c**) hyperplasia scores; (**d**) ACF scores; (**e**) microadenoma scores; (**f**) macroadenoma (low); (**g**) macroadenoma (high); (**h**) macroadenoma (low and high); (**i**) adenocarcinoma values. The extent of histological pathology was evaluated by a medical technologist. The values are the means ± SD (*n* = 5); ^##^ *p* < 0.01 and ^###^ *p* < 0.001 when compared to Con; * *p* < 0.5, ** *p* < 0.01, and *** *p* < 0.001 when compared to WD + AOM/DSS.

We scored the infammation, hyperplasia, and tumorigenicity levels based on the results shown in Figure 4a. It can be seen from the data in Figure 4b,c, that the inflammation and hyperplasia levels were elevated, whereas treatment with ASA, CPT, and orlistat significantly mitigated these levels in WD-driven CAC mice. The tumorigenicity extent is shown in Figure 4d–i. WD + AOM/DSS significantly accelerated tumor development compared to that of the Con group. In contrast, treatment with ASA, CPT, and orlistat significantly attenuated the development and progression of CRC.

### 3.5. Treatment with Orlistat Suppressed the Upregulation of STAT3 in WD-Driven CAC Mice

**Figure 5:**
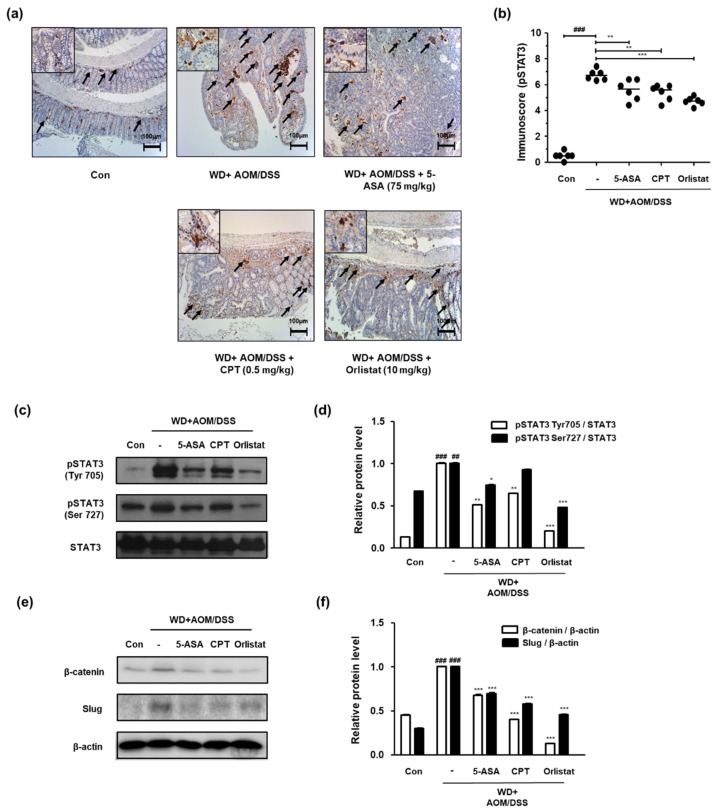
Effect of orlistat on the upregulation of STAT3 in WD-driven CAC mice. (**a**) Immunoreactivity of STAT3 Tyr705 was observed in colon tissues; (**b**) immunoscores for STAT3 Tyr705 in the colon of each group. Western blotting was conducted to detect the expressions of (**c**) pSTAT3 Tyr705 and Ser727; (**d**) relative protein levels for pSTAT3 Tyr705 and Ser727; (**e**) β-catenin and slug expressions; (**f**) β-catenin and slug relative protein levels. Relative protein levels calculated based on the internal control, STAT3, and β-actin. ^##^ *p* < 0.01 and ^###^ *p* < 0.001 when compared to Con; * *p* < 0.05, ** *p* < 0.01, *** *p* < 0.001 when compared to WD + AOM/DSS.

We confirmed the phosphorylation of STAT3 and the protein expression of STAT3 and STAT3-related genes in order to probe the effects of orlistat on the STAT3 signaling pathway in WD-driven CAC mice. Figure 5 shows that the expression of p-STAT3 Tyr 705 was significantly upregulated, whereas treatment with ASA, CPT, and orlistat significantly downregulated it in WD-driven CAC mice. We also found that the phosphorylation of STAT3 at the Tyr705 and Ser727 residues had increased in the WD + AOM/DSS group, but treatment with orlistat significantly repressed phosphorylation at these residues (Figure 5c,d). Similarly, the WD + AOM/DSS group showed less overexpression of β-catenin and slug protein expression, which is related to epithelial–mesenchymal transition (EMT) signaling. However, treatment with ASA, CPT, and orlistat significantly suppressed STAT3 and EMT-related protein expression (Figure 5e,f).

### 3.6. Treatment with Orlistat Inhibited the Activation of NF-κB in WD-Driven CAC Mice

**Figure 6:**
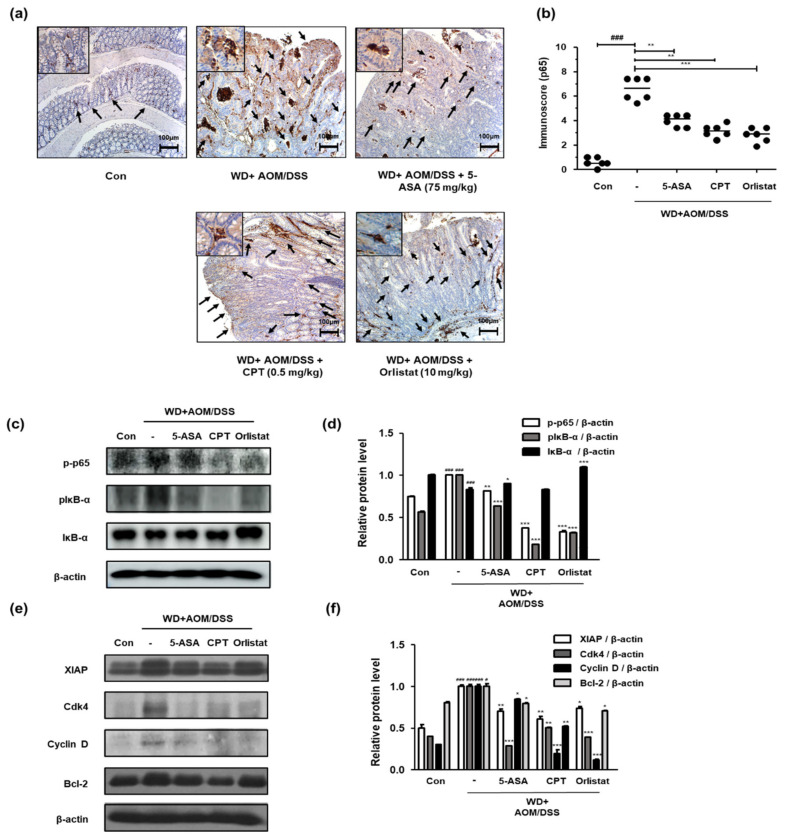
Effect of orlistat on NF-κB p65 target protein levels in WD-driven CAC mice. (**a**) Immunoreactivity of NF-κB p65; (**b**) estimated immunoscores for NF-κB p65; (**c**) p-p65, IκB, and pIκB expression; (**d**) p-p65, IκB, and pIκB relative protein expression levels; (**e**) NF-κB-related genes in colonic tissue were evaluated by western blot analysis; (**f**) XIAP, Cdk4, Cyclin D, and Bcl-2 relative protein levels. Relative protein expression levels were calculated by densitometric analysis and normalized to β-actin. ^#^ *p* < 0.05 and ^###^ *p* < 0.001 when compared to Con; * *p* < 0.05, ** *p* < 0.01, and *** *p* < 0.001 when compared to WD + AOM/DSS.

We confirmed the activation and expression of NF-κB and NF-κB-associated genes to identify the involvement of the NF-κB signaling pathway in the treatment of WD-driven CAC mice by orlistat. Figure 6a shows that WD-fed CAC mice exhibited a dramatic increase in NF-κB p65 protein expression. In contrast, treatment with ASA, CPT, and orlistat significantly suppressed the expression of NF-κB p65 in colonic tissues. Correspondingly, the p-p65 and pIκB protein levels were higher in the WD + AOM/DSS group than in the Con group, whereas the IκB protein level was lower in the WD + AOM/DSS group. In contrast, treatment with ASA, CPT, and orlistat significantly normalized these levels (Figure 6c,d). Furthermore, we observed that WD + AOM/DSS increased the XIAP, Cdk4, cyclin D, and Bcl-2 protein levels, whereas treatment with orlistat repressed the NF-κB p65-relative protein levels (Figure 6e,f).

## 4. Discussion

Colorectal cancer cases are mostly sporadic and usually caused by somatic mutations. Very often, CRC arises from prolonged IBD. Interestingly, although hereditary cases are rarely preceded by chronic inflammation, CRC can be prevented and delayed by anti-inflammatory medications, such as 5-ASA and aspirin [26,27]. These findings indicate a role for inflammation in carcinogenesis and offer a therapeutic approach when developing anti-inflammatory drugs for CAC therapy. Epidemiological and experimental studies have suggested that WD-induced obesity and obesity-mediated inflammation lead to increased CRC risk [28,29]. It has also been suggested that the inhibition of WD-driven CAC may be associated with anti-adipogenic activity, indicating a possible link between obesity and CAC [30]. In this study, WD consumption was estimated as a possible risk factor for inflammation-mediated CRC progression (Figure 1 and Table 4, Table 5 and Table 6). Although our results showed a decrease in fat weight in the WD/AOM + DSS group, cancer-related weight loss is an unavoidable result of the tumor stage, and the effects of WD consumption are considered valid.

The AOM/DSS-induced chemical injury model is one of the most widely used non-genotoxic CAC models. As human CAC is mainly caused by chronic exposure to low quantities of environmental mutagens [31], the mice were administered a single, low dose of AOM to initiate the process and repeated treatment with DSS to induce colitis. The AOM/DSS model supports the theory that inflammation is important in IBD-related colon carcinogenesis [32]. The results from our study demonstrate a strong and consistent association between obesity-induced inflammation and CAC in a WD-fed AOM/DSS-induced CAC mouse model (Figure 1 and Figure 2). As positive controls, we used two different chemical compounds: 5-ASA, which is a commonly used oral drug for the treatment of IBD and confers immunosuppressive effects, such as prostaglandin restriction and pro-inflammatory cytokine inhibition [5], and CPT, which is a topoisomerase inhibitor that causes DNA strand breaks that induce apoptosis in treated cells and has a high efficacy for the treatment of gastrointestinal cancer [33]. Our previous study showed that tumor progression decreased significantly following treatment with 5-ASA and CPT in a WD-driven CAC mouse model, suggesting a link between obesity-related inflammation and colon tumor formation [34].

Based on this evidence, we hypothesized that the anti-obesity drug orlistat represses WD-driven CAC. Previous studies have reported a risk of CRC after treatment with orlistat [35], although other studies have suggested that orlistat is not associated with excess CRC risk and that treatment with orlistat suppresses the proliferation of colon cancer cells [36,37]. Despite the controversy surrounding the effects of orlistat, recent studies have confirmed that orlistat has adaptive effects, such as anti-atherogenic and anti-NAFLD effects [38,39]. Additionally, it has been noted that orlistat ameliorated testicular dysfunction by inhibiting NF-κB-mediated inflammation in WD-fed rats [40], which showed an interconnection between the NF-κB pathway and the mechanism associated with orlistat.

The results from this study indicate that the major effect of orlistat was to resolve WD-driven CAC through the inhibition of NF-κB and STAT3 (Figure 5 and Figure 6). Data from previous sources show that NF-κB activation induces the upregulation of cancer-related factors, which is a possible pathway for tumor cell growth [41]. Many researchers have argued that NF-κB and STAT3 do not mediate tumorigenesis individually, but mediate it through crosstalk [26,42], and the repression of these two proteins has the potential to prevent and/or treat CRC [43]. In addition, our previous study found that STAT3 was activated in the WD-driven CAC model, which indicates that STAT3 is important for WD-mediated CAC aggravation [34].

Furthermore, treatment with orlistat significantly decreased the slug and β-catenin levels in colon tissues from WD-driven CAC mice (Figure 5). Generally, STAT3 and NF-κB activation enhances the aggressiveness and metastatic potential of cancer cells via the induction of EMT by the upregulation of EMT-inducing transcription factors, including slug [44,45]. In the AOM/DSS-induced CAC model, AOM aggravated DSS-induced inflammation and promoted CAC progression via the accumulation of β-catenin and the induction of EMT [46]. Therefore, orlistat might ameliorate WD-driven CAC via the inhibition of STAT3 and NF-κB, and/or NF-κB and STAT3-mediated EMT signaling.

## 5. Conclusions

The main findings can be summarized as follows: (1) WD consumption exacerbated AOM/DSS-induced CAC via inflammation, especially the activation of STAT3 and NF-κB in mice; (2) treatment with orlistat notably reduced the lethality rate and the formation of tumors in a WD-driven CAC mouse model; and (3) treatment with orlistat suppressed the aggressiveness of the cancer via the inhibition of STAT3, NF-κB, STAT3, and NF-κB-related gene expression. It is recommended that further experimental investigations are needed to assess the potential of orlistat as a potential chemopreventive agent against obesity-linked CAC.

## Figures and Tables

**Table 1:** Scores of histopathological inflammation.

Histological Parameters	Description	Score
Mucosa		
Epithelial cell	Prolonged epithelial cell or crypt	1
	Destruction of barrier	2
	Ulcer (30% < loss < 60%)	3
	Ulcer (loss > 60%)	4
Immune cell	Infiltration (mild)	1
	Infiltration (moderate)	2
	Infiltration (severe)	3
**Submucosa**		
Immune cell	Infiltration (mild)	1
	Infiltration (moderate)	2
	Infiltration (severe)	3

**Table 2:** Scores of histopathological hyperplasia.

Histological Parameters	Description	Score
Mucosa		
Non-dysplastic Epithelium	Mild (less than twofold) crypt length	1
	Intense crypt length with hyperchromatic CEC	2
Dysplastic Epithelium	Dysplastic epithelial region (legion < 20%)	1
	Dysplastic epithelial region (20% < legion < 50%)	2
		
	Dysplastic epithelial region (50% < legion < 90%)	4

**Table 3:** The extent of tumorigenicity.

Histological Parameters
Aberrant crypt foci (ACF)
Microadenoma
Macroadenoma (low grade)
Macroadenoma (high grade)
Macroadenoma (low and high grade)
Adenocarcinoma

**Table 4:** Inflammation scores.

Mucosa				
	**Con**	**WD**	**AOM/DSS**	**WD + AOM/DSS**
**Epithelial cells**	0	0.5	2	2.5
**Immune cell**	0.5	0.5	1.5	2
**Submucosa**				
**Immune cell**	0.5	0.5	1	1.5
**Total inflammation score**	1	1.5	4.5	6

Histologic assessment was performed on H&E slides. The values are the means ± SD (*n* = 6).

**Table 5:** Hyperplasia scores.

Mucosa				
	**Con**	**WD**	**AOM/DSS**	**WD + AOM/DSS**
**Non-dysplasic** **Epithelium**	0.5	0.5	3	6
**Dysplastic** **Epithelium**	1	1	1.5	4
**Total hyperplasia score**	1.5	1.5	4.5	10

Histologic assessment was performed on H&E slides. The values are the means ± SD (*n* = 6).

**Table 6:** Tumorigenicity scores.

Group	ACF	Microadenoma	Low-Grade Macroadenoma	High-Grade Macroadenoma	Adenocarcinoma
Con	0	0	0	0	0
WD	0	0	0	0	0
AOM/DSS	7	6	4	2	1
WD + AOM/DSS	10	9	6	7	1

## Data Availability

The data that support the findings of this study are available from the corresponding author upon reasonable request.

## Supplementary Materials

The following are available online at https://www.mdpi.com/article/10.3390/cells10082060/s1. Scheme S1: Scheme of the WD-propelled CAC mice model.
