# Supplementary figures and images for: Anti-Obesity Drug Orlistat Alleviates Western-Diet-Driven Colitis-Associated Colon Cancer via Inhibition of STAT3 and NF-κB-Mediated Signaling
Source: Cells. 2021 Aug 11;10(8):2060. doi: 10.3390/cells10082060 (PMC8394553; doi:10.3390/cells10082060)

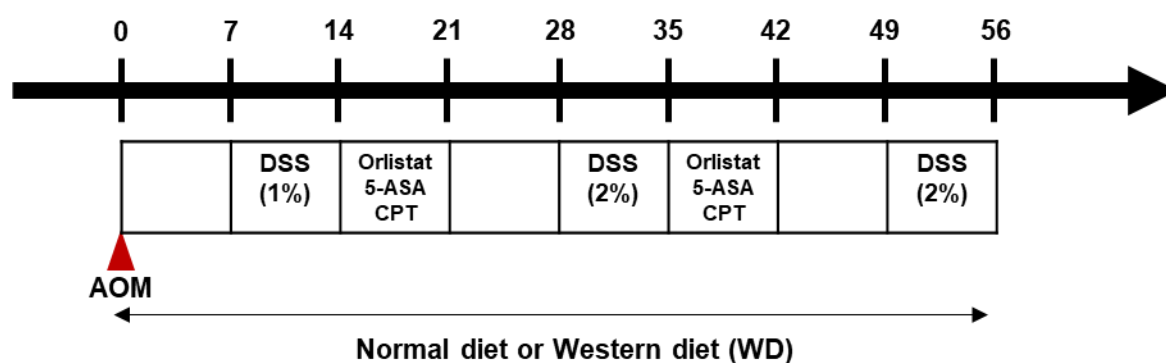

**Scheme S1.** Scheme of the WD-driven CAC mice model.

Supplement: Supplementary file 1 [file cells-10-02060-s001.zip › cells-1318166-supplementary.pdf]
